# Radiographic findings after radial head replacement

**DOI:** 10.1186/s13018-026-07103-9

**Published:** 2026-07-15

**Authors:** Gopal R. Lalchandani, Matthew Cherches, Ryan T. Halvorson, Akshara B. Vykunta, Lisa L. Lattanza, Nicolas H. Lee, Utku Kandemir

**Affiliations:** 1 UCSF Department of Orthopedic Surgery, 500 Parnassus Avenue, MU 320-W, San Francisco, CA 94143 USA; 2 UCSF School of Medicine, 513 Parnassus Ave S-245, San Francisco, CA 94143 USA; 3 Yale Department of Orthopaedics and Rehabilitation, 800 Howard Ave, New Haven, CT 06519 USA; 4 Department of Orthopaedic Surgery, University of California, San Francisco, 2540 23rd Street, Bldg 7, 3rd Floor, San Francisco, CA 94110 USA

**Keywords:** Radial head arthroplasty, Radiolucency, Osteolysis, Stress shielding, Heterotopic ossification, Radiocapitellar arthritis, Elbow

## Abstract

**Background:**

Radial head arthroplasty (RHA) has become the preferred treatment for displaced radial head and neck fractures that cannot be fixed. While good short-term results are reported, mid- to long-term radiographic findings such as loosening, stress shielding, and radiocapitellar arthritis are not uncommon. The aim of this study was to analyze and quantify radiographic findings after primary RHA. We hypothesized high rates of radiographic changes could be appreciated at mid-term follow-up.

**Methods:**

Patients at a single institution who underwent RHA between 2006 and 2019 with at least six months of radiographic follow-up were included. Associated injuries, fracture pattern, implant design, radiolucency, stress shielding, radiocapitellar arthritis, and heterotopic ossification were evaluated. Radiographic reviews were performed independently by two fellowship-trained orthopedic surgeons. Regression analysis was used to assess the relationship between demographics, clinical characteristics, and radiographic findings and reoperation.

**Results:**

Sixty-three elbows amongst 62 patients with a mean age of 47 (range 15–83) met inclusion criteria, with an average follow-up of 748.8 days (range 230–4554 days). At mid-term follow-up, 82.5% of patients had at least one radiographic finding, most of which were mild; stress shielding was present in 58.7% and radiolucency in 27.0%. In regression analysis, implant type was not associated with an increased odds for radial head specific reoperation. Age, follow-up duration, fracture pattern, and associated ligamentous injuries were not associated with SS, arthritis, HO, or radiolucency. Male gender was associated with a lower degree of radiocapitellar arthritis (estimate − 0.40; 95% CI − 0.72 to − 0.08), as was the use of bipolar implants (estimate − 0.6; 95% CI − 1.11 to − 0.09).

**Conclusion:**

Radiographic changes are frequently observed by mid-term follow-up after RHA, although most are mild and do not necessarily indicate clinical failure. Male gender and the use of bipolar implants may be protective against radiographic radiocapitellar arthritis. However, the clinical implications of these radiographic findings remain to be determined.

**Level of evidence:**

III.

**Type of study:**

Prognostic.

## Background

Radial head fractures represent the most common bony injury around the elbow and account for nearly 30% of all elbow fractures [1]. The goal of surgery following such injuries is to restore stability to the elbow while maintaining the relationship between the radius and distal humerus and ulna to allow for normal elbow and forearm kinematics. Consequently, multi-fragmentary radial head and neck fractures not amenable to open reduction and stable internal fixation are most commonly treated with radial head arthroplasty (RHA). Though trials have demonstrated a clear advantage of RHA over open reduction internal fixation in cases of complex multi-part radial head fractures [2], the long-term outcomes of these patients remain understudied.

While good short-term results are reported, long-term radiographic sequelae have been observed following primary radial head replacement [3, 16]. Such findings include evidence of loosening, stress shielding, heterotopic ossification (HO), and radiocapitellar arthritis. Importantly, these radiographic abnormalities do not uniformly correlate with clinical failure, and the clinical relevance of many radiographic findings after RHA remains controversial. However, existing literature on these radiographic findings after RHA is typically limited to small series [4, 5]. Chanlalit et al. reported a 63% rate of stress shielding after RHA; however, this was in a series of only 26 patients [1]. Similarly, Bershback et al. found noteworthy rates of osteolysis and HO formation after RHA, but again, this was in a series of 27 patients [6]. Given the rates of radiographic changes noted on long-term follow-up across prior smaller series, we aimed to investigate if this was the case in a larger cohort of patients.

Thus, the goal of this study was to analyze and quantify radiographic changes after primary radial head replacement. Additionally, we sought to identify the rate of RHA failure as well as the risk factors for subsequent surgery. Through examination of a larger study population, we hypothesized that radiographic findings could be appreciated earlier than previously described, by mid-term follow-up.

## Materials and methods

All study activities were approved by our institutional review board. A retrospective review of patients who underwent primary RHA for acute radial head and/or neck fractures between 2006 and 2019 at two centers were identified. All patients managed at two hospitals across a single academic institution, with at least six months of radiographic follow-up were included. Revision RHA patients including conversion from prior open reduction internal fixation to RHA were excluded. The primary outcomes studied were radiographic abnormalities at latest follow-up and subsequent surgery, including radial head-specific reoperation. Secondary analyses included interobserver reliability of the radiographic classification systems and exploratory multivariable analyses of factors associated with radiographic abnormalities and reoperation.

Associated bony or ligamentous injuries, the type of radial head implants and associated procedures were recorded. Most recent follow up visits with radiographs were used to identify the length of follow-up. Two fellowship-trained orthopedic surgeons independently conducted a radiographic review of each patient using multiple classification systems and their results were averaged.

The Johnston modification of the Mason classification was used to identify fracture patterns on preoperative radiographs [7]. Post-operative radiographs were used to confirm implant designs labeled in the operative reports. Radiographs at the most recent follow up were utilized to identify the presence of radiographic abnormalities. The Popovic classification was used to evaluate radiolucency around the implant and involves the division of the periprosthetic cortex of the proximal radius into seven zones to document radiolucent lines [6]. Radiolucency was considered present if at least one Popovic zone demonstrated a radiolucent line. The Chanlalit classification was used to evaluate stress shielding, and is defined as: Stage I, cortical bone thinning; Stage IIa, partially exposed stem; Stage IIb, circumferentially exposed stem; Stage III, mechanical or impending failure [1]. For stress shielding, any stage greater than 0 was considered present, regardless of severity. Radiocapitellar arthritis was graded using the Broberg-Morrey classification, which is defined as Grade 0, normal joint; Grade 1, slight joint-space narrowing with minimum osteophyte formation; Grade 2, moderate joint-space narrowing with moderate osteophyte formation; and Grade 3, severe degenerative change with gross destruction of the joint [8]. For radiocapitellar arthritis, any grade greater than 0 was considered present, regardless of severity. Finally, heterotopic ossification was evaluated using a binary modification of the Brooker classification based on the presence or absence of HO [9]. In this study, a patient was deemed to have HO present if both reviewers assigned a Brooker grade I or higher. The average score between the two reviewers was reported for each classification.

The interobserver reliability between teams for each of the rating systems was quantified using an unweighted Kappa for binary and categorical classification systems, a Spearman coefficient for ordinal classification systems, and a two-way intraclass correlation coefficient for continuous classification systems. Interobserver reliability was classified as *slight* (0–0.2), *fair* (0.21–0.40), *moderate* (0.41–0.60), *substantial* (0.61–0.80), and *almost perfect* (0.81–1.00) in accordance with previous literature [10].

Age- and gender-controlled multivariable regression models were used to analyze the relationship between fracture pattern (Johnston modification of Mason classification), implant type, and follow up duration on post-operative radiographic findings. A multivariable logistic regression was used to assess the effect of demographics, follow-up duration, implant type, injury severity (based on Johnston modification of Mason classification), SS, and radiolucency on odds of subsequent surgery (all causes). A similar analysis was performed to evaluate the effect of age, gender, follow-up duration, and implant type on radial head-specific reoperation. For multivariable analysis, implant types were grouped into broader design categories because the number of patients with several individual implant types was too small for stable subgroup estimates. Given the limited number of reoperation events, particularly radial head-specific reoperations, these multivariable analyses should be considered exploratory.

## Results

63 elbows amongst 62 patients with a mean age of 47 (range 15–83) met inclusion criteria, with an average follow-up of 749 days (range 170–4554 days). Of the 62 patients, 35 were male and 27 were female. 42 elbows (66.7%) sustained additional ligamentous injuries with radial head/neck fractures. 34 had unipolar anatomic press-fit, 9 had unipolar smooth stem, 8 had bipolar, and 12 had nonanatomic unipolar press-fit implants. 13 patients (13 elbows, 21%) underwent subsequent surgery (Table 1). 6 (9.5%) of the 13 subsequent surgeries were due to radial head replacement failure (Table 2). 7 of the 13 subsequent surgeries were not for radial head specific complications. These revisions were HO excision (2 patients), lateral ulnar collateral ligament reconstruction (1 patient), cubital tunnel release (1 patient), and removal of hardware not involving the radial head prosthesis (3 patients).

**Table 1:** Cohort characteristics by any subsequent surgery

	Any subsequent surgery		No subsequent surgery		Total	*P*
	n	%	n	%		
Total	13	100	50	100		
Age						0.75
< 35	5	38.5	14	28	19	
35–60	5	38.5	21	42	26	
> 60	3	23.1	15	30	18	
Gender						0.72
Male	8	61.5	28	56	36	
Female	5	38.5	22	44	27	
Average follow up duration days (min–max)	880 (207–3535)		715 (118–4554)			
Follow up > 1 year	10	76.9	25	50		0.081
Implant type						0.17
Anatomic press fit (acumed)	10	76.9	24	48		
Non-anatomic press fit (biomet, integra, stryker, synthes*)	0	0	12	26		
Smooth stem (wright)	2	15.4	7	12		
Bipolar head (tornier)	1	7.7	7	14		
Injury severity						
Fracture pattern Average (Johnston & Mason) (SD)	2.8 (0.51)		3.1 (0.49)			0.17
Associated Ligamentous Injury	7	53.8	35	70		0.27

The non-anatomic press fit implants by Synthes are recalled due to failures. This implant was used in 2 elbows in the current study. Neither elbow underwent subsequent surgery. One patient had radiolucency and SS, the other patient had radiolucency and HO

**Table 2:** Cohort characteristics by radial head specific reoperation

	Total	Radial head reoperation		No reoperation		P
		n	%	n	%	
Total	63	6	100	57	100	
Age						1
< 35	19	2	33.3	17	29.8	
35–60	26	2	33.3	24	42.1	
> 60	18	2	33.3	16	28.1	
Gender						1
Male	36	3	50	33	57.9	
Female	27	3	50	24	42.1	
Average follow up duration days (min–max)		680 (207–1296)		756 (118–4554)		0.71
Follow up > 1 year	35	3	50	32	56.1	1
Implant type						0.069
Anatomic press fit (acumed)	34	5	83.3	29	50.9	
Non-anatomic (Synthes, Biomet, Integra, Stryker, Tornier)	12	0	0	12	21.1	
Smooth stem (wright)	9	1	16.7	8	14	
Bipolar head (tornier)	8	0	0	8	14	
Injury severity						
Fracture pattern average (Johnston & Mason Classification) (SD)		2.83 (0.51)		3.08 (0.49)		0.29
Associated Ligamentous Injury	42	2	33.3	40	70.2	0.089

Among all cases, both reviewers found 58.7% of patients had evidence of SS (Chanlalit I-III), 27.0% had evidence of radiolucency (at least one Popovic zone), 15.9% had HO, and 23.8% had evidence of radiocapitellar arthritis (Broberg-Morrey 1–3) (Table 3). These category-specific findings were not mutually exclusive and may have co-occurred within the same elbow. In total, 42 elbows (66.7%) had radiographic evidence of SS or radiolucency. At least one radiographic finding (HO, SS, arthritis, or radiolucency) was identified by both sets of reviewers in 52 elbows (82.5%), many of which represented low-grade changes according to the applied classification systems.

**Table 3:** Incidence of radiographic findings

Finding	Overall		Any subsequent surgery (all-cause)		Radial head reoperation		No subsequent surgery	
	n	%	n	%	n	%	n	%
Total	63	100	13	100	6	100	50	100
Radiolucency (Both at least one zone)	17	27.0	5	38.5	4	66.7	12	24.0
Stress Shielding (Both reviewers Chanlalit > 0)	37	58.7	10	76.9	5	83.3	27	54.0
Radiocapitellar Arthritis (Both reviewers Broberg-Morrey > 0)	15	23.8	4	30.8	2	33.3	11	22.0
Heterotopic Ossification (Both reviewers Brooker > 0)	10	15.9	2	15.4	1	16.7	8	16.0

In a multivariable binary logistic regression to predict the odds of subsequent surgery, non-anatomic implants were associated with decreased odds of all-cause subsequent surgery (OR 95% CI 0.00–0.60, *p* = 0.036, Table 4). However, when looking at radial head specific reoperation, implant type was not associated with odds of reoperation (Table 4). Age, gender, follow up duration, injury severity, SS, and radiolucency were not associated with odds of subsequent surgery or radial head specific reoperation (Tables 4, 5). Due to the small number (6) of revisions for radial head specific reasons, the multivariable model to predict revision of radial head arthroplasty was simplified to limit the number of predictor variables. Given the small numbers of radial head revisions across a variety of implants used, we were unable to associate any particular radial head implant type with increased risk of radial head loosening.

**Table 4:** Multivariable binary logistic regression to predict subsequent surgery

	OR	*P*
Age		
< 35	Reference	
35–60	0.03–1.67	0.13
> 60	0.04–3.66	0.39
Gender		
Female	Reference	
Male	0.66–22.43	0.18
Follow up > 1 year	0.86–29.45	0.08
Implant type		
Anatomic press fit (acumed)	Reference	
Smooth stem (wright)	0.06–4.11	0.49
Non-anatomic (Synthes, Biomet, Integra, Stryker, Tornier)	**0.00–0.60**	**0.04**
Injury severity		
Fracture pattern average (Johnston & Mason)	0.05–1.69	0.22
Associated ligamentous injury	0.04–1.47	0.11
Radiographic findings		
Stress shielding (Chanlalit)	0.34–2.28	0.82
Radiolucency (Popovic)—Proportion of Zones	0.08–23.09	0.78

**Table 5:** Multivariable binary logistic regression to predict radial-head-specific reoperation

	OR	95% CI	*P* value
Age			
< 35	1	Reference	
35—60	0.61	0.06–5.94	0.66
> 60	1.05	0.11–10.6	0.96
Gender			
Female	1	Reference	
Male	0.94	0.15–6.06	0.95
Follow up			
< 1 year	1	Reference	
> 1 year	0.75	0.12–4.68	0.75
Implant type			
Not acumed	1	Reference	
Acumed	5.21	0.74–105.17	0.15

In multivariable analysis to predict radiographic findings, age, follow-up duration, fracture pattern (Johnston modification of Mason classification), and associated ligamentous injuries were not associated with SS, radiolucency arthritis, or HO. Male gender was associated with a lower degree of radiocapitellar arthritis (estimate − 0.40; 95% CI − 0.72 to − 0.08, Table 6), but gender was not associated with SS, radiolucency, or HO. The use of bipolar head implants was also associated with a lower degree of radiocapitellar arthritis (estimate − 0.6; 95% CI − 1.11 to − 0.09, Table 6), but implant type was not associated with SS, radiolucency or HO.

**Table 6:** Multivariable regressions to predict radiographic characteristics

	Stress shielding (Chanlalit)				Radial-capitellar arthritis (Broberg Morrey)				Heterotopic ossification (Brooker)				Radiolucency (Popovic)			
	Linear/Gaussian				Linear/Gaussian				Binary/Binomial				Linear/Gaussian			
	Est	2.5%	97.5%	P	Est	2.5%	97.5%	P	Est	2.5%	97.5%	P	Est	2.5%	97.5%	P
Age																
< 30	Ref				Ref				Ref				Ref			
35–60	0.44	− 0.23	1.10	0.20	− 0.08	− 0.46	0.30	0.67	1.32	0.29	6.57	0.72	0.02	− 0.39	0.43	0.92
> 60	0.41	− 0.32	1.13	0.28	0.17	− 0.25	0.59	0.44	1.65	0.30	9.64	0.56	0.03	− 0.42	0.48	0.90
Gender																
Female	Ref				Ref				Ref				Ref			
Male	− 0.02	− 0.57	0.54	0.95	**− 0.40**	**− 0.72**	**− 0.08**	**0.02**	1.12	0.32	4.14	0.86	0.09	− 0.25	0.44	0.60
Follow Up > 1 year	− 0.14	− 0.71	0.43	0.63	0.18	− 0.15	0.50	0.30	1.00	0.29	3.57	1.00	0.06	− 0.30	0.41	0.75
Injury severity																
Fracture pattern Average (Johnston & Mason Classification)	− 0.09	− 0.64	0.46	0.75	0.14	− 0.17	0.46	0.37	1.62	0.47	6.06	0.45	0.03	− 0.31	0.38	0.84
Associated Ligamentous Injury	0.00	− 0.58	0.59	0.99	0.30	− 0.04	0.64	0.09	0.81	0.23	3.09	0.75	− 0.17	− 0.53	0.20	0.37
Implant type																
Anatomic press fit (Acumed)	Ref				Ref				Ref				Ref			
Non-anatomic press fit (Biomet, Integra, Stryker, Synthes)	− 0.66	− 1.37	0.06	0.08	0.40	− 0.01	0.81	0.06	0.45	0.06	2.31	0.38	− 0.24	− 0.68	0.20	0.30
Bipolar head (Tornier)	− 0.34	− 1.22	0.54	0.45	**− 0.60**	**− 1.11**	**− 0.09**	**0.02**	0.34	0.02	2.85	0.38	− 0.21	− 0.75	0.33	0.45
Smooth Stem (Wright)	− 0.61	− 1.41	0.18	0.14	0.32	− 0.14	0.77	0.18	1.99	0.37	10.30	0.41	0.34	− 0.15	0.83	0.18

Ref: reference, Est: estimate. Statistically significant findings shown in bold

Regarding interobserver reliability, agreement between the two teams of reviewers was moderate (0.41–0.60) for radiolucency (Popovic classification). Agreement was *fair* (0.21–0.40) for HO (using a binary modification of the Brooker classification) and for SS (Chanlalit classification). Agreement was *slight* (0.00–0.20) for radiocapitellar arthritis (Broberg-Morrey classification).

## Discussion

The current study identified the rate of radiographic abnormalities amongst 63 elbows in 62 patients who underwent RHA for acute fracture. While recent larger series on the topic have been published outside of the orthopedic literature [11], our data almost double the numbers presented in prior classic series on the topic by Berschback et al. and Chanlalit et al.

While both of these series had longer durations of follow-up of at least one year, with a mean follow-up of 33 months for both series, our series only required a minimum of 6 months of follow-up with a shorter mean follow-up of 25 months (747 days). Our series revealed significant rates of stress shielding (58.7%) and radiolucency (27%), with 66.7% of elbows with evidence of stress shielding or radiolucency and 82.5% of elbows having at least one radiographic abnormality. These data indicate that radiographic abnormalities may occur sooner than suggested by previous literature [1, 6]. In addition, 9.5% of elbows required revision surgery for radial head prosthesis loosening.

On multivariable analysis, the use of anatomic implants was associated with an increased odds of all-cause subsequent surgery. However, this association did not persist when the analysis was restricted to radial head-specific reoperation. This suggests that the observed association may reflect factors other than implant-specific failure, including differences in injury complexity, surgeon decision-making, or non–implant-related causes of subsequent surgery. Prior literature similarly suggests that reoperation after RHA is multifactorial, with common reasons for revision including symptomatic loosening, stiffness, and persistent pain [15].

We found a lower rate of radiocapitellar arthritis associated with bipolar implants and also in male patients on multivariable analysis for predictors of radiographic findings. The former finding may be attributed to the fact that bipolar implants can distribute motion and shear forces between inner and outer bearings, thus sparing the capitellum from erosion and wear [13]. Similarly, the association between male patients and lower rates of radiographic radiocapitellar arthritis may be due to typically higher bone density in male patients [14]. However, we also found that interobserver reliability for radiocapitellar arthritis was *slight* using the Broberg-Morrey classification. More broadly, the slight-to-moderate interobserver agreement observed across several radiographic classification systems may have reduced the precision of radiographic outcome classification overall. Therefore, analyses examining associations between radiographic findings and patient or implant factors should be interpreted cautiously.

Intraoperatively, differences in implant geometry and implantation technique may affect how reliably an implant can be positioned. Although anatomic implants were associated with greater odds of all-cause subsequent surgery, this study was not designed to determine whether this reflected implant design, technical difficulty of implantation, or other unmeasured factors. Radiographic findings may be related to implant design and/or surgical technique, which warrants future study. Importantly, these findings should not be automatically interpreted as clinical failure, particularly in the absence of patient-reported outcome measures. In our study, stress shielding and radiolucency were common radiographic findings, yet neither was associated with odds of subsequent surgery or radial head-specific reoperation on multivariable analysis. This suggests that radiographic abnormalities may not necessarily correlate with clinical outcomes; a patient with such findings may not have corresponding symptoms or functional limitations. Moreover, radiographic abnormalities may not be progressive over time and may stabilize short of any adverse clinical outcomes. On the other hand, one must be cautious regarding the long-term clinical implications of radiographic abnormalities. We advise both long-term clinical and radiographic follow-up of patients if any radiographic findings are present.

Another important finding of this study is the slight to moderate interobserver reliability when using the aforementioned classification systems for loosening, stress shielding, heterotopic ossification, and radiocapitellar arthritis. Even after converting the Brooker classification to a binary classification system to detect either the presence or absence of HO, the interobserver reliability remained *fair.* This is consistent with previously published findings that suggest interobserver reliability of classification systems for radiographic complications after RHA is less than substantial [12]. This limited agreement reduces the precision of radiographic outcome classification and weakens the strength of inferences that can be drawn from these findings. As such, both the reported prevalence of radiographic abnormalities and analyses examining associations between radiographic findings and patient, implant, or surgical factors should be interpreted cautiously. This indicates an area of further study to help optimize classifications for detecting radiographic abnormalities after RHA.

Limitations of our study include the retrospective nature of our study. While we attempt to draw correlations between radiographic and clinical outcomes in our study, we are limited by the data that have already been gathered and are unable to gather additional functional or patient-reported outcomes to help clarify the significance of these radiographic findings. Additionally, indications for RHA, implant choices, and the need for subsequent surgery were all at the discretion of the attending surgeon of each case. There were thus no pre-determined criteria for revision surgery prior to conducting this study. A further limitation is that radiographic assessment was based on the most recent available follow-up radiographs rather than serial postoperative imaging; therefore, we could not determine the timing of onset or progression of individual radiographic abnormalities, which warrants future study. Another limitation of this study is the small sample size. This may predispose us to type II error, as we were unable to identify a correlation between radiographic findings and clinical outcomes in this study—potentially due to a small sample size and low number of reoperation events. Accordingly, analyses of predictors of subsequent surgery, especially radial head-specific reoperation, should be interpreted as exploratory. Further studies to prospectively follow a larger number of patients and measure clinical outcomes can help address these limitations.

## Conclusion

This study demonstrates radiographic abnormalities are frequently observed by mid-term follow-up after RHA for acute fractures. Male gender and the use of bipolar implants may be protective factors against radiographic radiocapitellar arthritis. However, the clinical implications of these radiographic findings require further study.

## Data Availability

The data analyzed during the study were obtained from Zuckerberg San Francisco General Hospital and the University of California, San Francisco and included all patients treated with radial head arthroplasty with adequate post-op follow-up during the study period. The datasets analyzed are not publicly available to keep Protected Health Information secure but are available from primary author on reasonable request.

## Abbreviations

RHA: Radial head arthroplasty
SS: Stress shielding
HO: Heterotopic ossification
